# Challenges with Using Primer IDs to Improve Accuracy of Next Generation Sequencing

**DOI:** 10.1371/journal.pone.0119123

**Published:** 2015-03-05

**Authors:** Johanna Brodin, Charlotte Hedskog, Alexander Heddini, Emmanuel Benard, Richard A. Neher, Mattias Mild, Jan Albert

**Affiliations:** 1 Department of Microbiology, Tumor and Cell Biology, Karolinska Institutet, Stockholm, Sweden; 2 Max Planck Institute for Developmental Biology, Tuebingen, Germany; 3 Unit for Support, Swedish Institute for Communicable Disease Control, Stockholm, Sweden; 4 Department of Clinical Microbiology, Karolinska University Hospital, Stockholm, Sweden

## Abstract

Next generation sequencing technologies, like ultra-deep pyrosequencing (UDPS), allows detailed investigation of complex populations, like RNA viruses, but its utility is limited by errors introduced during sample preparation and sequencing. By tagging each individual cDNA molecule with barcodes, referred to as Primer IDs, before PCR and sequencing these errors could theoretically be removed. Here we evaluated the Primer ID methodology on 257,846 UDPS reads generated from a HIV-1 SG3Δenv plasmid clone and plasma samples from three HIV-infected patients. The Primer ID consisted of 11 randomized nucleotides, 4,194,304 combinations, in the primer for cDNA synthesis that introduced a unique sequence tag into each cDNA molecule. Consensus template sequences were constructed for reads with Primer IDs that were observed three or more times. Despite high numbers of input template molecules, the number of consensus template sequences was low. With 10,000 input molecules for the clone as few as 97 consensus template sequences were obtained due to highly skewed frequency of resampling. Furthermore, the number of sequenced templates was overestimated due to PCR errors in the Primer IDs. Finally, some consensus template sequences were erroneous due to hotspots for UDPS errors. The Primer ID methodology has the potential to provide highly accurate deep sequencing. However, it is important to be aware that there are remaining challenges with the methodology. In particular it is important to find ways to obtain a more even frequency of resampling of template molecules as well as to identify and remove artefactual consensus template sequences that have been generated by PCR errors in the Primer IDs.

## Introduction

Ultra-deep pyrosequencing (UDPS) is one application of 454 next-generation sequencing (NGS) that has been used for identification of minority variants, for example in HIV populations resistant to antiretroviral drugs [1–5]. The accuracy of UDPS is limited by the relatively high error rate of the 454 sequencing technology and by errors introduced during cDNA synthesis and PCR amplification prior to 454 sequencing [3, 6–9]. 454 sequencing errors primarily involve insertions and deletions (indels) in homopolymeric regions, i.e. stretches of identical nucleotides in the target sequence [3, 6–9]. The impact of these errors can be partially alleviated by using post-sequencing data cleaning procedures [7, 10, 11]. In contrast, errors introduced during cDNA synthesis and PCR are usually single nucleotide substitutions, in particular transitions, are difficult to correct by post-sequencing data cleaning procedures [7]. Importantly, this latter type of errors is also relevant to other NGS platforms like Illumina, Ion Torrent and Pacific Biosciences, that currently is replacing the 454 platform that we used in the present study.

In order to overcome the problem of *in vitro* copying errors introduced during reverse-transcription and PCR confounding identification of variants, Jabara and colleagues developed a method for reducing errors in identification of HIV variants using template re-sampling [12]. In this approach, the reverse-transcription primer includes a random sequence tag such that each template receives a unique Primer ID. Similar approaches have been used in other NGS applications [13–15]. We have developed a method similar to that described in [12] which employs Primer IDs as template-specific molecular tags. In our Primer ID NGS methodology the Primer ID consists of 11 randomized nucleotides that are integrated in the primer for cDNA synthesis. This gives each individual cDNA molecule a specific identification tag, i.e. Primer ID, which is maintained during PCR and NGS. This allows for sorting of the NGS reads based on their Primer ID and for construction of consensus sequences for each original template molecule, which we call “consensus template sequence”. Theoretically these consensus template sequences should accurately represent the original cDNA molecule. Another advantage of the Primer ID NGS approach is that it allows enumeration of how many template molecules have been sequenced, which eliminates the need to measure input template numbers and reduces the risk of unintentional overestimation of number of templates and sequencing depth [15].

Here we describe challenges that we have encountered in the application of the Primer ID methodology on the 454 platform. This includes poor recovery of consensus template sequences due to skewed resampling, over-estimation of the number of sequenced templates due to PCR-induced mutations in Primer IDs, and erroneous consensus template sequences due to hotspots for UDPS errors. Researchers need to be aware of these challenges if they want to apply the exciting PID methodology on the 454 platform as well as other NGS platforms.

## Materials and Methods

### Samples

The HIV-1 SG3Δenv plasmid was used as a control to investigate the accuracy of the Primer ID UDPS method. The plasmid was also used previously [1, 7] and is available at the NIH AIDS Research and Reference reagent Program under catalogue no. 11051 and the sequence of the parent plasmid pSG3.1 is available in Genbank under accession no. L02317. Plasma samples from three HIV-infected patients (A, B and C) were also investigated. The patient samples analyzed for minority resistance in this study were selected from a large longitudinal study on transmitted drug resistance in Sweden 2003–2010 [16].

The research in this study was approved by the Regional Ethics Review Board in Stockholm (Dnr 2007/1533) and conducted according to the Declaration of Helsinki. The patients had given written or oral consent for participation. The use of oral consent, as an alternative to written consent, was permitted to minimize selection biases due to patient drop-out because some ethnic groups of participants were known to be willing to take part in the study, but reluctant to provide written consent. Both written and oral consent was documented in the patient records.

### RNA extraction, cDNA synthesis and PCR amplification using Primer IDs

**Fig 1:**
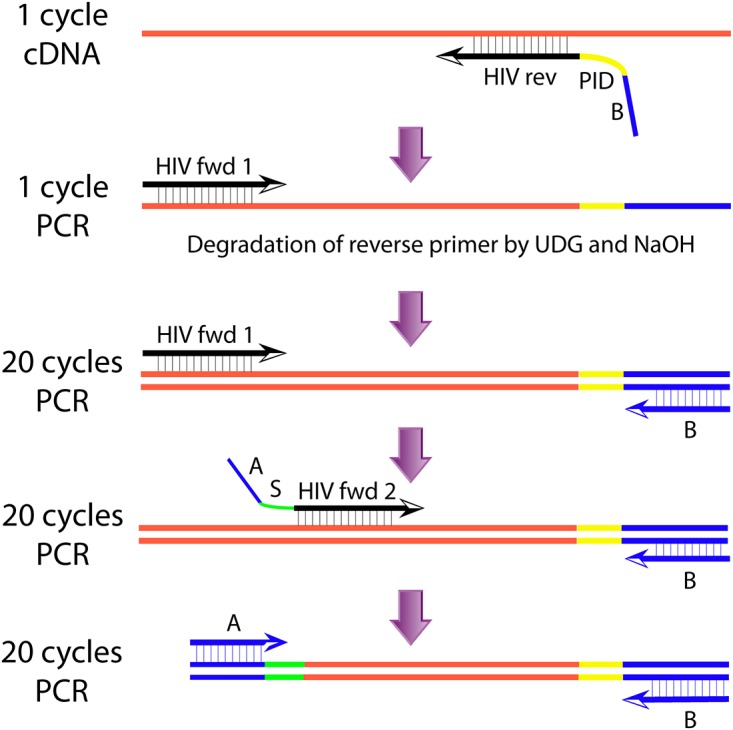
Schematic illustration of the Primer ID UDPS method. HIV rev, HIV-specific region (reverse complement). PID, Primer ID region. B, Region corresponding to 454 adaptor B. UDG, Uracil-DNA glycosylase. HIV fwd 1, HIV-specific forward primer (JA269). A, Region corresponding to 454 adaptor A. S, Sample tag. HIV fwd 2, HIV-specific region. Red, HIV-1 RNA. Yellow, Primer ID sequence. Blue, adaptor. Green, patient sample tag. For details on primers see S1 Fig.

The Primer ID UDPS method is illustrated in Fig. 1. The methods for RNA extraction, cDNA synthesis and semi-nested PCR amplification were modified from a published protocol [1]. The PCR targets a 167-nucleotide fragment (nucleotide position 3059 to 3226 in the HxB2 reference sequence according to the Sequence Locator Tool available at www.hiv.lanl.gov), which fully encompasses amino acid positions 169 to 223 in the HIV reverse transcriptase (RT). The primers are given in S1 Fig.

### RNA extraction

HIV RNA was extracted and purified with the RNeasy Lipid Tissue Mini Kit (Qiagen, Hilden, Germany) using the QIAvac 24 vacuum manifold protocol (Qiagen, Hilden, Germany). The amount of plasma used for extraction was 1000 μL. RNA was eluted in 40 μl RNase free water. The HIV-1 copy number, i.e. the actual number of viral templates subjected to UDPS, was quantified for each sample using an in-house limiting dilution PCR method adapted from Brinchmann *et al*. [1, 17]. This PCR was performed with nested primers JA269, JA272, JA329 and JA331 (see S1 Fig.), which target the same 167-nucleotide fragment as the PCR used for 454-sequencing (see below). The PCR conditions were also the same. The limiting dilution PCR was performed in 10 replicates with 5-fold dilution steps starting with a 1/10 dilution. The HIV-1 copy number was calculated using the Poisson distribution formula.

### Introduction of Primer IDs

The Primer ID was introduced using primer CH331, which contained an HIV-specific region, a Primer ID region and a region corresponding to the 454 adaptor B sequence (Fig. 1 and S1 Fig.). This primer, and all other primers, was standard desalted and purchased from IDT (Integrated DNA Technologies, Leuven, Belgium). The Primer ID consisted of 11 randomized nucleotides for a total of 4,194,304 combinations, which introduced a unique sequence tag into each cDNA molecule. The CH331 primer had thymidine bases replaced by uracil bases to allow downstream degradation by uracil-DNA glycosylase (UDG). For the SG3Δenv plasmid DNA the Primer ID was introduced by using the CH331 primer in a single PCR cycle with Platinum Taq High Fidelity (Life Technologies, Stockholm, Sweden) and the PCR conditions below. For the patient plasma samples the Primer ID was introduced by cDNA synthesis using the CH331 primer. For denaturation and priming extracted RNA (8 μl) and primer was incubated at 65°C for 5 min followed by a short incubation at 4°C. Next, Thermoscript (Life Technologies, Stockholm, Sweden) was added and cDNA synthesized by incubation at 42°C 15 min, 50°C 30 min, 85°C 5 min and finally 4°C according to the according to the manufacturer’s instructions. cDNA synthesis was done in five parallel reactions to allow reverse transcription of all available RNA.

### Generation of dsDNA and degradation of cDNA synthesis primer

The cDNA (5 μl) was converted into dsDNA by a single PCR cycle using HIV-specific primer JA269 (Fig. 1). This and subsequent PCR reaction mixtures contained: 1 unit of Platinum Taq High Fidelity, 1x High Fidelity PCR buffer, 2.0 mM MgSO_4_, 0.2 mM of each dNTP (all reagents from Life Technologies, Stockholm, Sweden) and 0.2 μM of each primer (IDT) in a total volume of 50 μl. The PCR cycling profile was as follows: one initial denaturation step at 94°C for 20 s, annealing at 50°C for 20 s, and extension at 72°C for 90 s followed by a final 6-minute extension at 72°C. After this, the uracil-containing cDNA synthesis primer was degraded by incubation with 1 unit of UDG at 37°C for 30 min and 95°C for 10 min (for inactivation of UDG), and then by incubation with 0.18 M NaOH at 37°C for 10 min. Finally, the NaOH was neutralized by HCl. These reactions were done in 17 parallel tubes to allow synthesis of the complementary DNA strand for all cDNA molecules. After degradation of the cDNA primer the resulting dsDNA was concentrated using QIAamp DNA Mini Kit (Qiagen, Hilden, Germany) and eluted in 50 μl nuclease free water.

### Semi-nested PCR

The dsDNA was amplified in three semi-nested PCRs (Fig. 1). The first PCR was done with outer forward primer JA269 and reverse primer B, which corresponds to the 454 adaptor B sequence. This PCR was carried out in 10 parallel reactions that subsequently were pooled to make all cDNA templates available for the following PCRs and 454 sequencing. The second PCR was done with the inner forward primer CH329, which had a HIV-specific region, a four nucleotide long sample-specific tag (to allow simultaneous sequencing of several samples) and the 454 adaptor A sequence (Fig. 1 and S1 Fig.). The 454 adaptor B again was used as reverse primer. Finally, a third PCR was done with primers corresponding to 454 adaptors A and B. All three PCRs were done using 20 PCR cycles and the same PCR conditions as for generation of dsDNA (see above). A higher fidelity enzyme (Phusion) were tested, but selected against because it was found to be less efficient than Platinum Taq in amplifying the target sequence.

### 454 sequencing

Before UDPS, the PCR amplicons were purified using 1.4 volumes of Agencourt AMPure Beads (Beckman Coulter Genomics, Danvers, Massachusetts, US) and the DNA concentration and purity was determined using Qubit 2.0 Fluorometer (Life Technologies, Carlsbad, California, US). In addition, the Agilent 2100 Bioanalyzer (Agilent Life Science, Santa Clara, California, US) was used to verify the quality and length of the amplicons. After quality controls, PCR amplicons from the different samples were pooled and sequenced in both forward and reverse direction on the 454 Life Sciences platform (GS-FLX Titanium, Roche Applied Science) according to the manufacturer’s instructions.

### Sanger sequencing

The PCR products from all samples were also subjected to population Sanger sequencing (ABI Prism 3100) using the Big Dye terminator cycle sequencing kit according to recommendations by the manufacturer (Life technologies, Foster City, California, US).

### Data analysis

We used in-house Python scripts to analyze the 454 sequence data. The quality of each read was controlled using the 454 quality scores. If any of the quality scores of the first 12 bases in either the 3’ or 5’ end was < 20 or if the average quality score of the read was < 20 the read was removed. Remaining reads were aligned to the 3′ and 5′ HIV specific primers and checked for a correct sample tag and a Primer ID of at least 10 bases, and separated according to the sample tag. Reads that did not align well to the primers (more than 3 mismatches or indels) or lacked sample tags or Primer IDs were discarded. A disproportionate number of reads lacked the last nucleotide of the read (i.e. the last nucleotide of the Primer ID for forward reads and the last nucleotide of the sample tag for reverse reads). This appeared to be due to excessive trimming of the 454 adapter sequences by the 454 software. For this reason the first nucleotide of the sample tags as well as the last nucleotide of the Primer IDs was not considered. This trimming effectively shortened the Primer ID from 11 to 10 nucleotides, which is inconsequential since the degeneracy of the remaining Primer ID is still high enough to allow sparse sampling of the space of IDs (4^10^ = 1,048,576). Liang et. al. recently showed that up to 50,000 RNA templates can be detected with 95% probability using a 10 bases long Primer ID [18]. Similarly, the sample tags could be distinguished based on the last three nucleotides. In addition, 12,728 reads (113 reads from the clone and 516, 10,517, 1,582 from patients A-C respectively) that differed by more than seven nucleotides from the Sanger sequence of the respective sample were discarded because such divergent reads are likely contaminants. In fact, most of the discarded reads were contaminants from the clone.

For each sample, reads with the same Primer ID were aligned using Muscle [19]. We created strict majority-rule consensus template sequences from the reads with Primer IDs that were observed at least three times. Consensus sequences were multiple-aligned with Muscle and the BioEdit software was used to inspect sequence alignments. Next, we aligned each Primer ID to all less abundant Primer IDs to detect Primer IDs that could have arisen by a single mutation or indel from an abundant class of Primer IDs. In case the template consensus sequences of two neighboring Primer IDs are similar, it is likely that the less abundant Primer ID was generated by mutation from the more abundant ID. Alternatively, the less abundant ID might represent a valid template. Given the typical error rate of PCR, we merged the corresponding sets of reads if they differed at less than four positions in template consensus sequences (other thresholds yield similar results). If one of the two Primer IDs was found in less than three reads, the reads were tested individually. Consensus sequence construction was repeated for these “corrected” sets of reads.

### Statistics

To quantify artefactual generation of Primer IDs by mutations during PCR and sequencing, we needed to develop a statistical method that was insensitive to possible amplification biases and sequencing artefacts. To this end, we constructed an upper bound for the number of nearest neighbors given that we begin the experiment with *m* template molecules. Preferential sampling of particular sequences and mutations in barcodes will increase the number of nearest neighbors—which is what we want to detect.

The probability that two randomly sampled Primer IDs differ at *k* nucleotides is given by *P*(*k*) = Binom(*k*,10,0.75). Since the number of available Primer IDs (4^10^) is much larger than the number of template molecules *m*, the number of Primer IDs that differ at *k* sites from a given Primer ID in a sample of size *m* is Poisson distributed with mean *P*(*k*)(*m-1*), at least for small *k*. Hence the probability that we observe *n* Primer IDs that have more than *r* neighbors at distance *k* = 1 is binomially distributed with success probability *p* = Sum(Poisson(*s*, *P*(*k*)(m-1)), *s*>*r*). We measured this number *n* in each of the samples and tested the null hypothesis of random Primer ID sampling using the binomial test with *p* and r = 1,3,6. Note that this argument assumes that the number of *k* = 1 neighbors can be independently assessed for each Primer ID, which is an accurate approximation given the high dimensionality of the space.

## Results

### Characteristics of UDPS data

We performed Primer ID UDPS on the SG3Δenv plasmid clone and three patient plasma samples (Table 1). The number of input template molecules was determined using limiting dilution analysis. After removal of low-quality reads we obtained a total of 229,232 UDPS reads with identifiable sample tags and Primer IDs, which were retained for further analyses.

**Table 1:** Characteristics of samples and sequence data.

				No. of consensus template sequences		
Sample	No. of input template molecules	No. of reads	No. of reads with PIDs observed at least 3 times	Uncorrected	Corrected for PID substitutions	Corrected for PID substitutions and indels
Clone	10,000	47,387	47,225	97	23	14
Patient A	18,900	104,597	102,192	2,103	2,000	1,786
Patient B	24,000	57,159	56,317	263	200	184
Patient C	5,850	20,089	19,816	120	103	99

### Skewed resampling of Primer IDs

We constructed consensus template sequences for reads with Primer IDs that were observed three or more times. Despite high numbers of input template molecules, the number of consensus template sequences was low (Table 1). This was especially pronounced for the SG3Δenv clone for which we used 10,000 input molecules, but obtained as few as 97 consensus template sequences corresponding to less than 1% of available template molecules. Similar results were obtained for the patient samples where the number of consensus template sequences corresponded to less than 2% and 3% of available template in two patients and 12% in the third patient.

**Fig 2:**
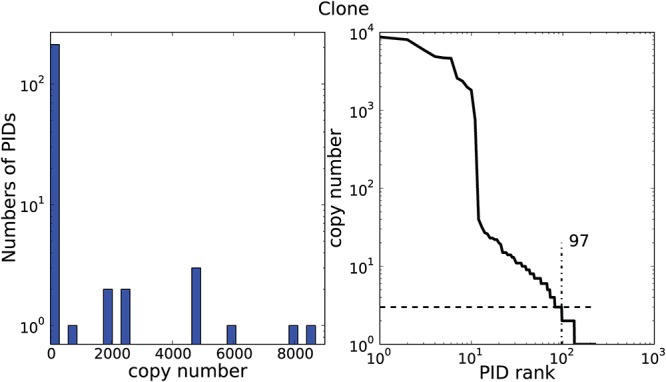
Distribution of Primer ID resampling for clone SG3Δenv. Panel A shows the copy number distribution of Primer IDs (see methods). Eleven Primer IDs were sampled more than 100 times, while the remaining Primer IDs were sampled rarely. Panel B shows the rank distribution of Primer ID copy number indicating the threshold of at least three sequences per Primer ID necessary for consensus sequence construction. In this sample, 97 Primer IDs were above this threshold. More than a hundred Primer IDs were sampled only once or twice.

The low number of consensus template sequences appeared to be due to skewed frequency of resampling of individual template molecules. This is illustrated for the clone in Fig. 2. Some template molecules were resampled more than 9,000 times, whereas other template molecules were resampled just a few times. In fact, 162 (0.34%) reads could not be used to construct consensus template sequences because their Primer IDs were only observed once or twice. Similar data were obtained for the patient samples (S2 Fig., S3 Fig. and S4 Fig.). We also examined if the Primer IDs from frequently resampled template molecules had any special features compared to less frequently resampled template molecules with respect to nucleotide at the starting/ending position, nucleotide composition, melting temperature, GC content, homopolymeric regions, sequence repeats, and sequencing direction. No such differences were observed (data not shown), but the power to detect differences was relatively low given that our analyses were based on a limited number of PIDs. No identical Primer IDs was found in two different samples.

### Overestimation of number sequenced templates due to PCR errors in Primer IDs

**Fig 3:**
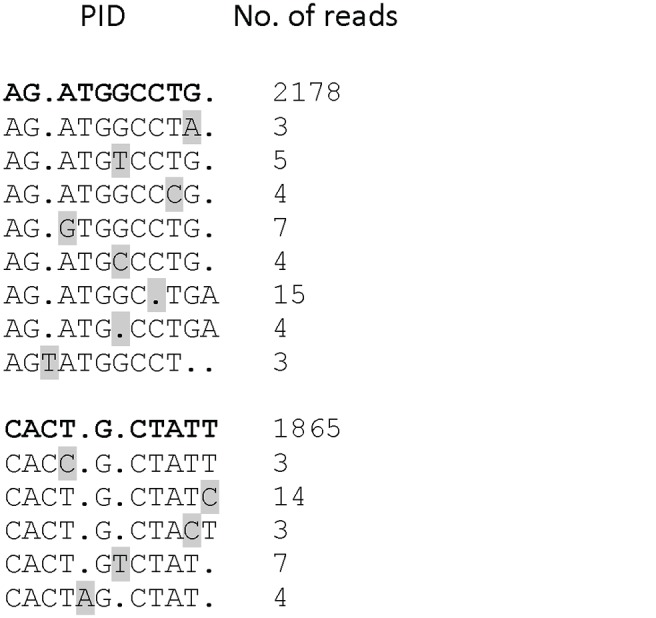
Examples of experimental errors in Primer IDs. Alignment of two “families” of closely related Primer IDs consisting of one frequently re-sequenced “parent” Primer ID (in bold) and several infrequently re-sequenced “offspring” Primer IDs with single nucleotide substitutions, insertions or deletions (in grey).

Despite the low recovery of consensus template sequences we found that some Primer IDs differed by only a single nucleotide. This was unexpected given to the high degeneracy of the Primer IDs, i.e. 4^10^ = 1,048,576. Furthermore, we identified groups or “families” of closely related Primer IDs. Typically there was one “parent” Primer ID that had been re-sequenced many times and one or more closely-related “offspring” Primer IDs that had been re-sequenced much fewer times (Fig. 3). We hypothesized that these offspring Primer IDs might have been artificially created from genuine Primer IDs by PCR or UDPS errors introduced after labeling in the cDNA synthesis step. If so, each such family of parent and offspring Primer IDs would have been derived from the same original template molecule and should be counted only once. To test this hypothesis we calculated the probability to observe a certain number of Primer IDs with more than 1, 3 or 6 “neighbors” that differ at only one position, based on the assumption that Primer IDs are randomly sampled from the pool of available Primer IDs (see methods). Since we cannot control amplification biases of different Primer IDs, we conservatively evaluate this probability for a sample size given by the number of template molecules rather than the number of Primer IDs recovered (S1 Table). In all cases, except patient B, we find that the observed number of Primer IDs with more than three neighbors is larger than what would be expected based on random Primer ID sampling with *p*-values below 10^-4^. Consequently, two Primer IDs that differ at one of ten positions are more likely to be a pair generated by PCR-induced mutation or UDPS error than independently sampled from pool of available Primer IDs. Assuming a sequencing error rate of 10^-3^ and a Primer ID length of 10, we expect one mutation in the Primer ID for every 100 reads. While sequencing errors will generate rare artefactual Primer IDs, PCR-induced mutations in early PCR cycles can give rise to abundant artefactual Primer IDs. Primer IDs with two or more non-identical positions are more likely to represent distinct template molecules.

### Sequencing errors despite use of Primer IDs

**Fig 4:**
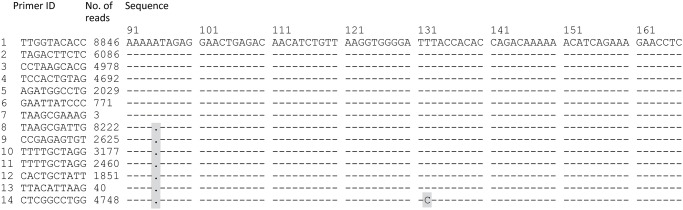
Errors in consensus template sequences. Alignment of 14 partial template consensus sequences from clone SG3Δenv. Errors compared to the correct clone sequence are highlighted.

The 14 corrected consensus template sequences from the SG3Δenv clone were considered to contain errors if they did not exactly match the published sequence of the clone, which was identical to our *de novo* Sanger population sequence. Surprisingly, only 7 of 14 (50%) consensus template sequences from the clone were correct (Fig. 4). All 7 incorrect consensus sequences contained an erroneous deletion of an adenosine (A) in a homopolymeric stretch of five A’s from position 91 to 95 of the amplicon. One of the incorrect template consensus sequences in addition had a T-to-C substitution error at position 132 (sequence #14 in Fig. 4).

To investigate the reason for the frequent deletion error at position 95, as well as the reason for the other sequencing error, we examined the reads that were used to build each of the erroneous consensus template sequences. We reasoned that if a mutation had been present among the original template molecules from the clone or had been introduced during cDNA synthesis, we would expect all reads from the template to have the mutation in question. If instead, an error had been introduced during PCR or UDPS, we would expect the mutation to be present in some, but not all, reads from the template. We found that the latter pattern was true for all 7 consensus template sequences with the deletion error at position 91–95 that had been re-sequenced more than 10 times and also for some that had been re-sequenced fewer times. Thus, these consensus template sequences were constructed from reads with and without the deletion error. Furthermore, we found that this homopolymeric stretch was a hotspot for UDPS errors because the deletion was present in 51% of all quality-filtered reads from the clone, which was not unexpected given the known problems with pyrosequencing of homopolymeric regions. Thus, it is not surprising that more than 50% of the reads from some templates contained this deletion, which resulted in an erroneous consensus template sequence. Due to the observed pattern, i.e. the UDPS error hotspot and consensus template sequences that were constructed from reads with and without the deletion, we could conclude that all deletion errors at position 95 were due to a systematic UDPS error. The T-to-C substitution error at position 132 in consensus sequence #14 was examined in the same way and was found to be present in approximately 95% of 4,748 reads from this template. This indicates that it had been introduced during cDNA synthesis (or was present among the templates of the clone).

## Discussion

In this paper we describe some important challenges that were encountered during evaluation of the Primer ID UDPS technology. This includes poor recovery of consensus template sequences due to skewed resampling, over-estimation of the number of sequenced templates due to PCR-induced mutations in Primer IDs and erroneous consensus template sequences due to hotspots for UDPS errors. The possibility of skewed resampling has previously been mentioned [12], but to our knowledge the other problems have not been reported. The 454 NGS platform is currently being replaced by newer NGS platforms such as Illumina, Ion Torrent and Pacific BioSciences. However, the Primer ID methodology is generic and can be used for these platforms. Furthermore, the methodology is not limited to HIV or virology, but has already been used also in other scientific projects [13–15]. Consequently, our findings are of relevance to all applications of Primer IDs in NGS.

A main finding was that the number of reads per template was highly skewed, which led to several downstream problems. First, the total number of template consensus sequences was much lower than expected because some templates were resampled thousands of times at the cost of other templates that instead were sampled too few times to construct a consensus sequence, or not at all. The high number of reads from certain templates also meant that we obtained artefactual template consensus sequences due to errors that were introduced in the Primer IDs after cDNA synthesis and labeling, i.e. during PCR and/or 454 sequencing. Skewing was most pronounced for the clone, but there was substantial skewing also in the patient samples. Similarly and probably related to degree of skewing, errors in Primer IDs were more frequent in the clone than in the patient samples. At this point it is unclear if there is a systematic difference between these two types of samples. We are currently adapting the Primer ID technology to newer NGS platforms to investigate this as well as the cause of the highly skewed resampling of available template molecules. We and others have shown good reproducibility in quantification of complex mixtures of HIV-1 variants using UDPS analysis without Primer IDs [20, 21]. This suggests that skewing is induced by the Primer ID itself or the long cDNA primer, because this is the major difference between standard UDPS and Primer ID UDPS. It is possible that the Primer IDs leads to differences in amplification efficiency of different templates, for instance due to higher or lower tendency for formation of hairpins or primer dimers. In this study we did not find any specific characteristics of frequently vs. less frequently resampled PIDs, but the power to detect such differences was suboptimal. However, unpublished data generated on the Ion Torrent platform indicate that nucleotide composition might be a contributing factor. We are investigating this issue as well as if designed Primer IDs may help to achieve more homogeneous resampling of template molecules. In studies that did not involve UDPS, Shiroguchi and coworkers have shown that designed single-molecule barcodes attached to each end of the template sequence may alleviate PCR amplification biases while retaining high degeneracy in the barcode [22]. Another approach would be to investigate whether the length of the Primer ID influences the evenness of resampling. However, shorter Primer IDs will also mean a reduced number of unique configuration combinations, which will make it more difficult to identify PCR-induced mutations in the Primer IDs. In addition, our primer for cDNA synthesis contained uracil bases, instead of thymidines, to allow for efficient downstream degradation of the primer. This was done because we found that significant amounts of primer remained after standard column purification, which could lead to relabeling of templates. We cannot exclude the possibility that the uracils may have impacted on template labeling and thereby skewing. Finally, we used nested PCR in our experiments because our 454-sequencing protocol required relatively high DNA input. Newer NGS platforms, and in particular Illumina, require much less input DNA. It is quite possible that skewing may be reduced by avoiding nested PCR and by using fewer PCR cycles. Newer NGS platforms may also allow for other changes in the experimental protocol.

By analyzing a clone we found that the Primer ID methodology is not yet completely accurate. Most of the remaining sequencing errors were deletions of one nucleotide in a homopolymeric region that was a hotspot for deletion errors, a well-known problem in pyrosequencing. When >50% of the reads from a template molecule contained this deletion error it resulted in an incorrect consensus template sequence. However, often errors of this type can be identified because the reads that build up the consensus template sequence will be a mixture of reads with and without the indel in question. This allows for automated bioinformatic identification and correction of errors of this type. Misincorporations in the cDNA synthesis or first PCR cycle are another source of errors in current Primer ID UDPS protocols, which only can be avoided by direct molecular tagging of the HIV-1 RNA molecules. Polymorphisms in the plasmid templates are a third possible source of apparent errors in our experiment.

In summary, the Primer ID methodology has the potential to provide highly accurate deep-sequencing. However, it is important to be aware that there are remaining challenges with the methodology. In particular it is important to find ways to obtain a more even frequency of resampling of template molecules as well as to identify and remove artefactual consensus template sequencing that have been generated by errors in the Primer IDs.

## Supporting Information

S1 Table Probability to observe a certain number of Primer IDs. (PDF)

S1 Fig Primers used for cDNA synthesis and PCR. For overview of method see Fig. 1.
(TIF)

S2 Fig Distribution of template resampling for patient sample A. Panel A shows the copy number distribution of Primer IDs (see methods). Panel B shows the rank distribution of Primer ID copy number indicating the threshold of at least three sequences per Primer ID necessary for consensus sequence construction.
(TIF)

S3 Fig Distribution of template resampling for patient sample B. Panel A shows the copy number distribution of Primer IDs (see methods). Panel B shows the rank distribution of Primer ID copy number indicating the threshold of at least three sequences per Primer ID necessary for consensus sequence construction.
(TIF)

S4 Fig Distribution of template resampling for patient sample C. Panel A shows the copy number distribution of Primer IDs (see methods). Panel B shows the rank distribution of Primer ID copy number indicating the threshold of at least three sequences per Primer ID necessary for consensus sequence construction.
(TIF)
