# Supplementary material for: Challenges with Using Primer IDs to Improve Accuracy of Next Generation Sequencing
Source: PLoS One. 2015 Mar 5;10(3):e0119123. doi: 10.1371/journal.pone.0119123 (PMC4351057; doi:10.1371/journal.pone.0119123)
Supplement: S1 Table — (PDF) [file pone.0119123.s001.pdf]

Table S1. Probability to observe a certain number of Primer IDs (PIDs) with more than 1, 3 or 6 "PID neighbors" that differ at only one position, based on the assumption that Primer IDs are randomly sampled from the pool of available Primer IDs (see methods). The sample size of PIDs was calculated based the actual number of template molecules as well as the actual number of recovered PIDs.

Sample	No. of templates	No. of PID neighbors (Observed; expected; p-value)		
		>1	>3	>6
Clone	10,000	118; 339; $<10^{-9}$	15; 2.2; $2x10^{-8}$	11; 0; $<10^{-9}$
	14	118; 0; $<10^{-9}$	15; 0; $<10^{-9}$	11; 0; $<10^{-9}$
Patient A	18,900	461; 1,943; $<10^{-9}$	17; 44; $6.94x10^{-6}$	0; 0.03; na
	1,786	461; 2.3; $<10^{-9}$	17; 0; $<10^{-9}$	0; 0; na
Patient B	24,000	170; 3,628; $<10^{-9}$	63; 129; 0.112	14; 0.2; $<10^{-9}$
	184	170; 0; $<10^{-9}$	63; 0; $<10^{-9}$	14; 0; $<10^{-9}$
Patient C	5,850	64; 73; 0.29	15; 0.17; $<10^{-9}$	2; 0; $<10^{-9}$
	99	64; 0; $<10^{-9}$	15; 0; $<10^{-9}$	2; 0; $<10^{-9}$

na, not applicable
