# Supplementary figures and images for: Challenges with Using Primer IDs to Improve Accuracy of Next Generation Sequencing
Source: PLoS One. 2015 Mar 5;10(3):e0119123. doi: 10.1371/journal.pone.0119123 (PMC4351057; doi:10.1371/journal.pone.0119123)

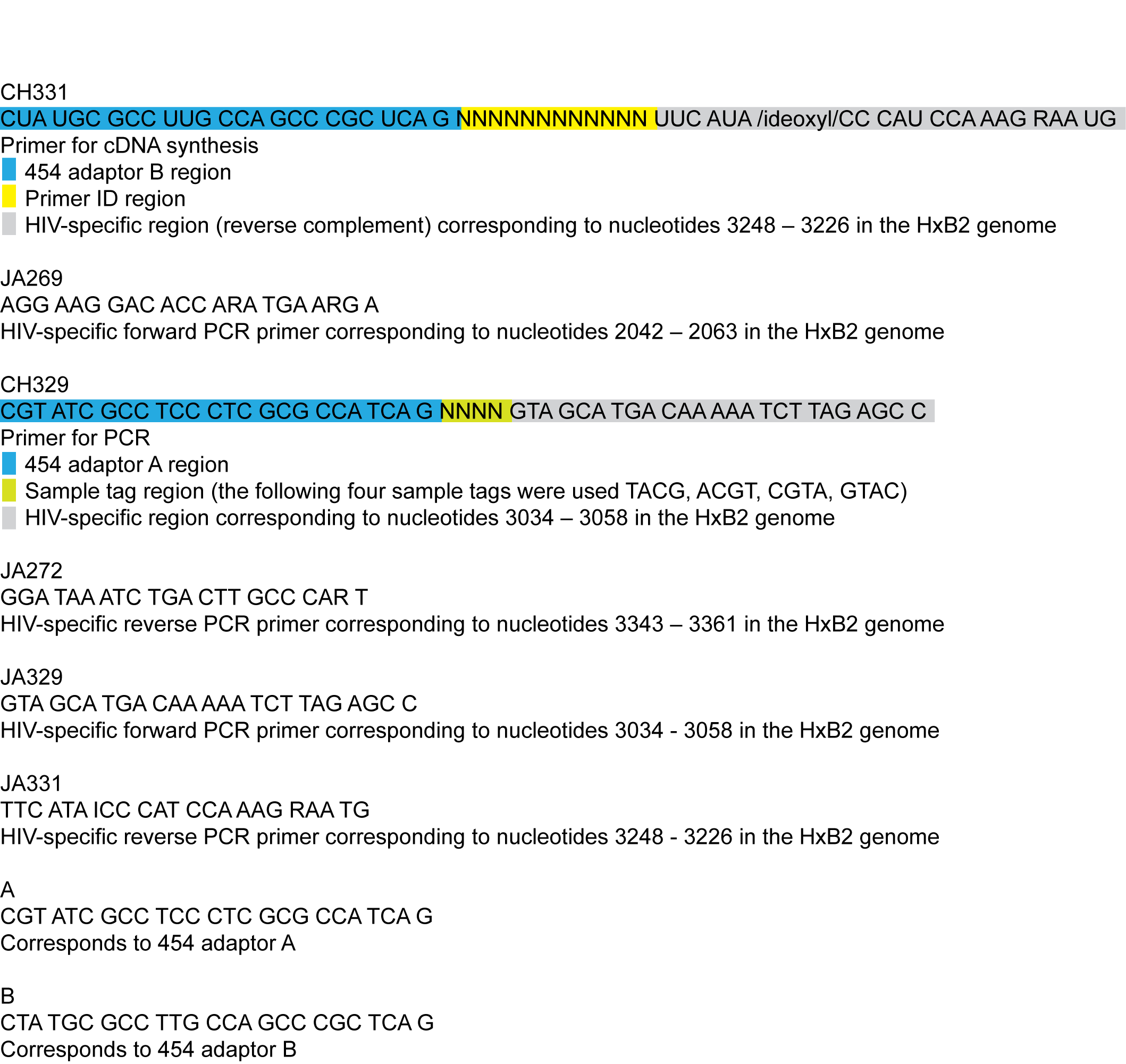

Supplement: S1 Fig — For overview of method see Fig. 1. (TIF) [file pone.0119123.s002.tif]

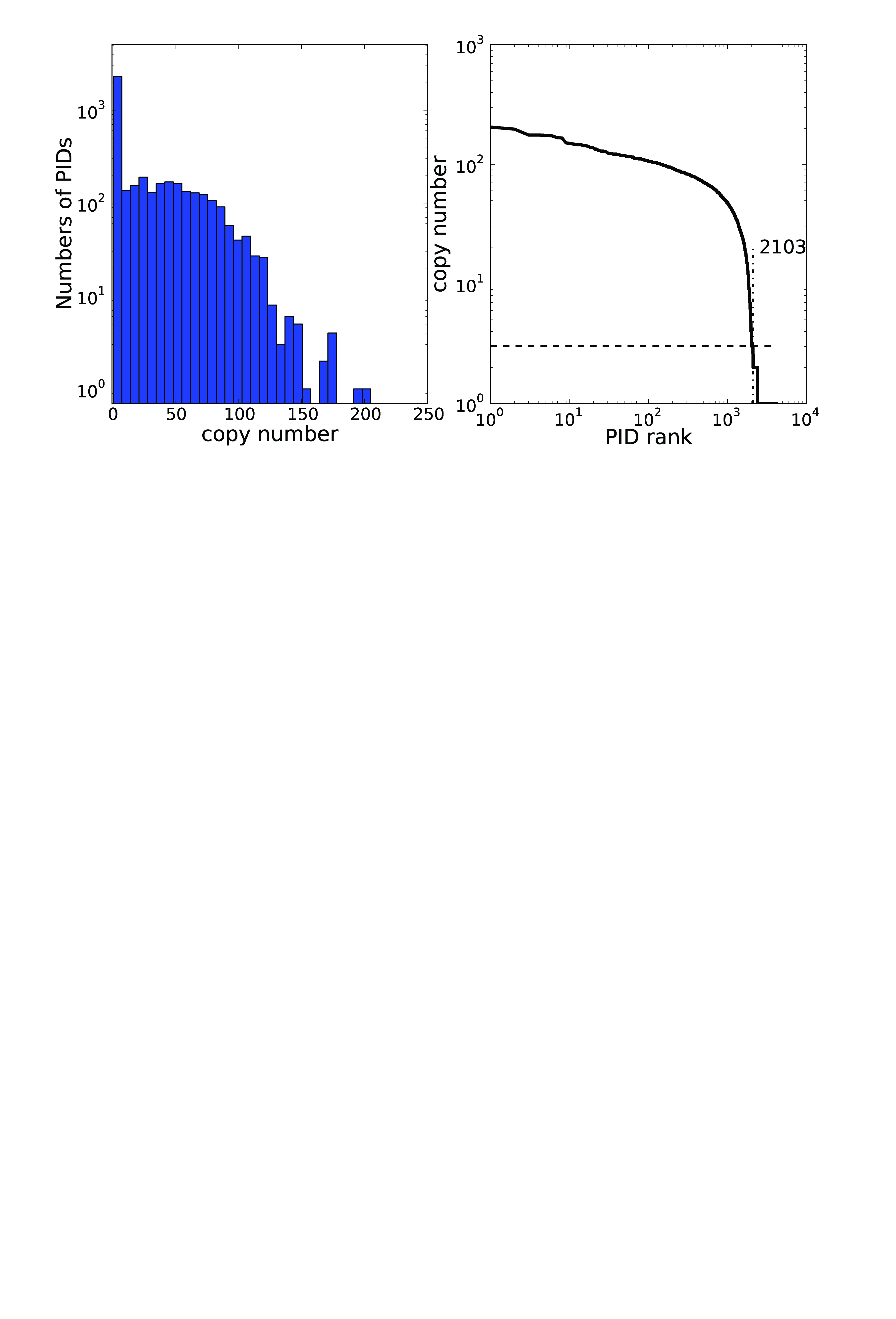

Supplement: S2 Fig — Panel A shows the copy number distribution of Primer IDs (see methods). Panel B shows the rank distribution of Primer ID copy number indicating the threshold of at least three sequences per Primer ID necessary for consensus sequence construction. (TIF) [file pone.0119123.s003.tif]

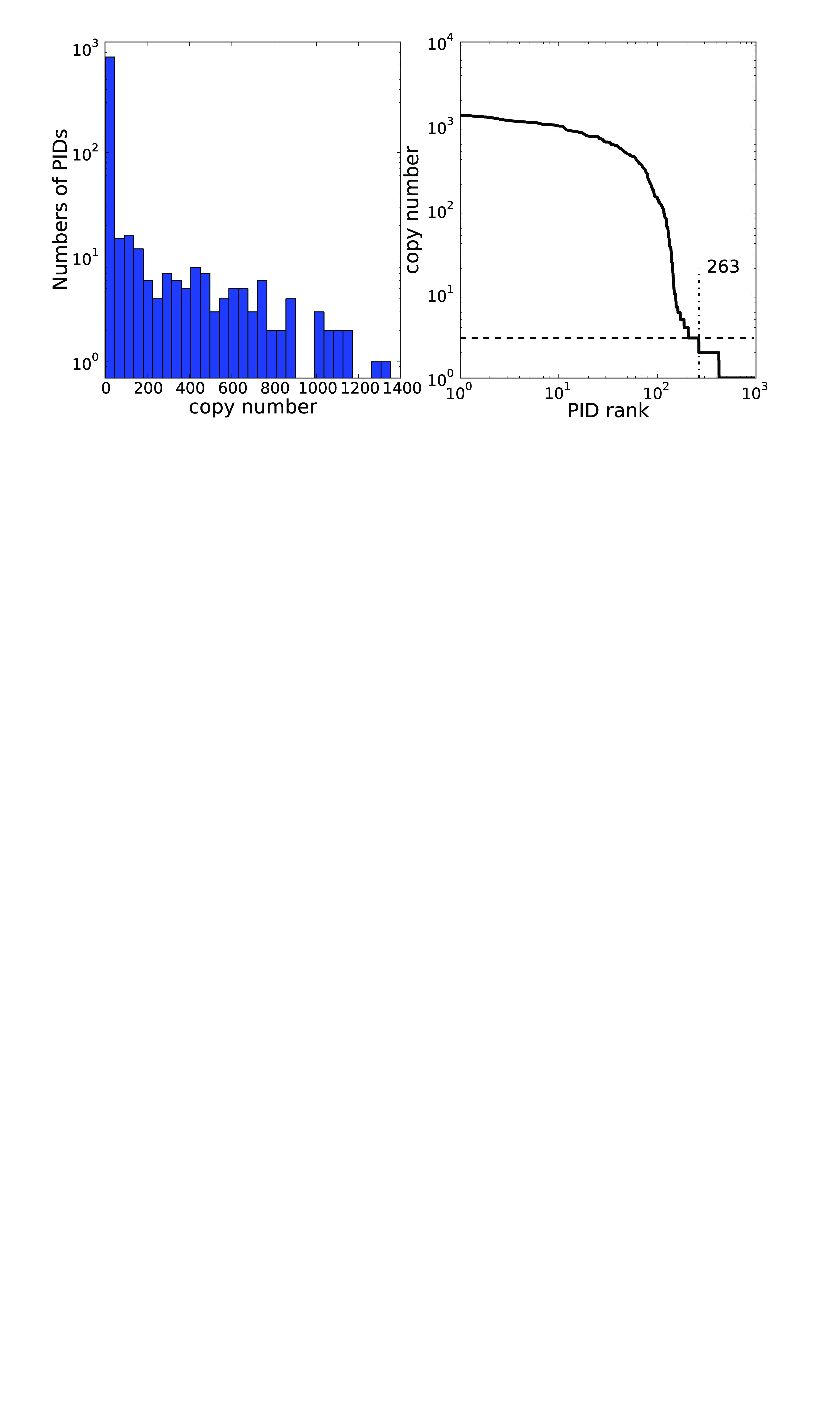

Supplement: S3 Fig — Panel A shows the copy number distribution of Primer IDs (see methods). Panel B shows the rank distribution of Primer ID copy number indicating the threshold of at least three sequences per Primer ID necessary for consensus sequence construction. (TIF) [file pone.0119123.s004.tif]

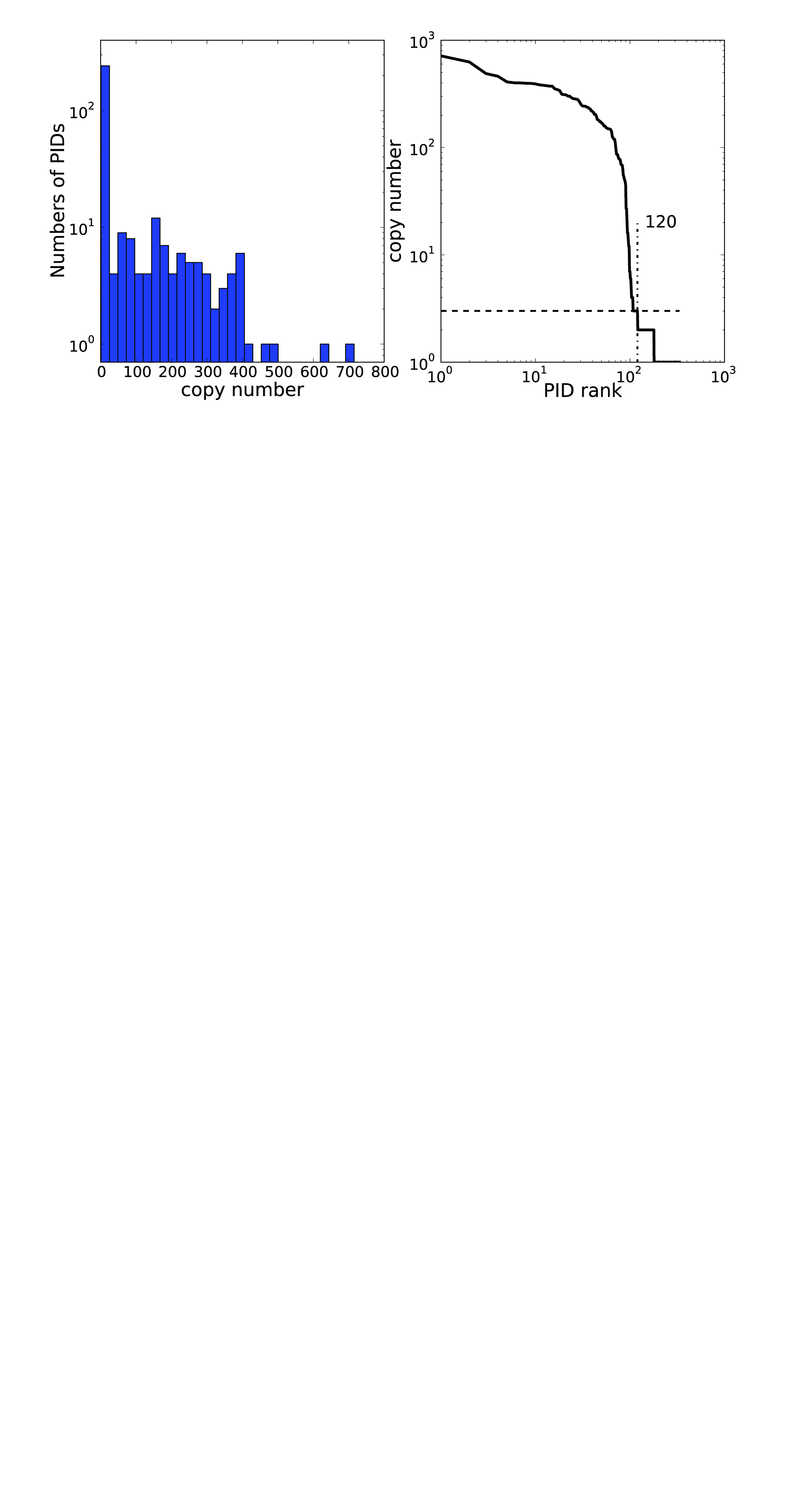

Supplement: S4 Fig — Panel A shows the copy number distribution of Primer IDs (see methods). Panel B shows the rank distribution of Primer ID copy number indicating the threshold of at least three sequences per Primer ID necessary for consensus sequence construction. (TIF) [file pone.0119123.s005.tif]
